# Supplementary figures and images for: Imbalance between Expression of FOXC2 and Its lncRNA in Lymphedema-Distichiasis Caused by Frameshift Mutations
Source: Genes (Basel). 2021 Apr 27;12(5):650. doi: 10.3390/genes12050650 (PMC8146868; doi:10.3390/genes12050650)

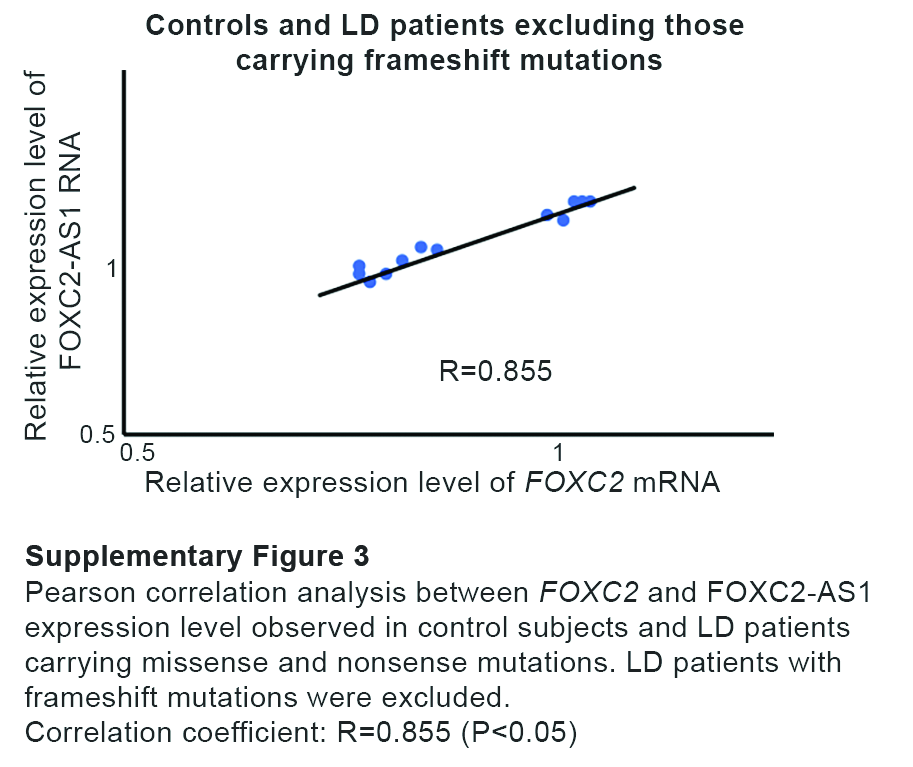

Supplement: Supplementary file 1 [file genes-12-00650-s001.zip › Supplemenary Figure 3.tif]

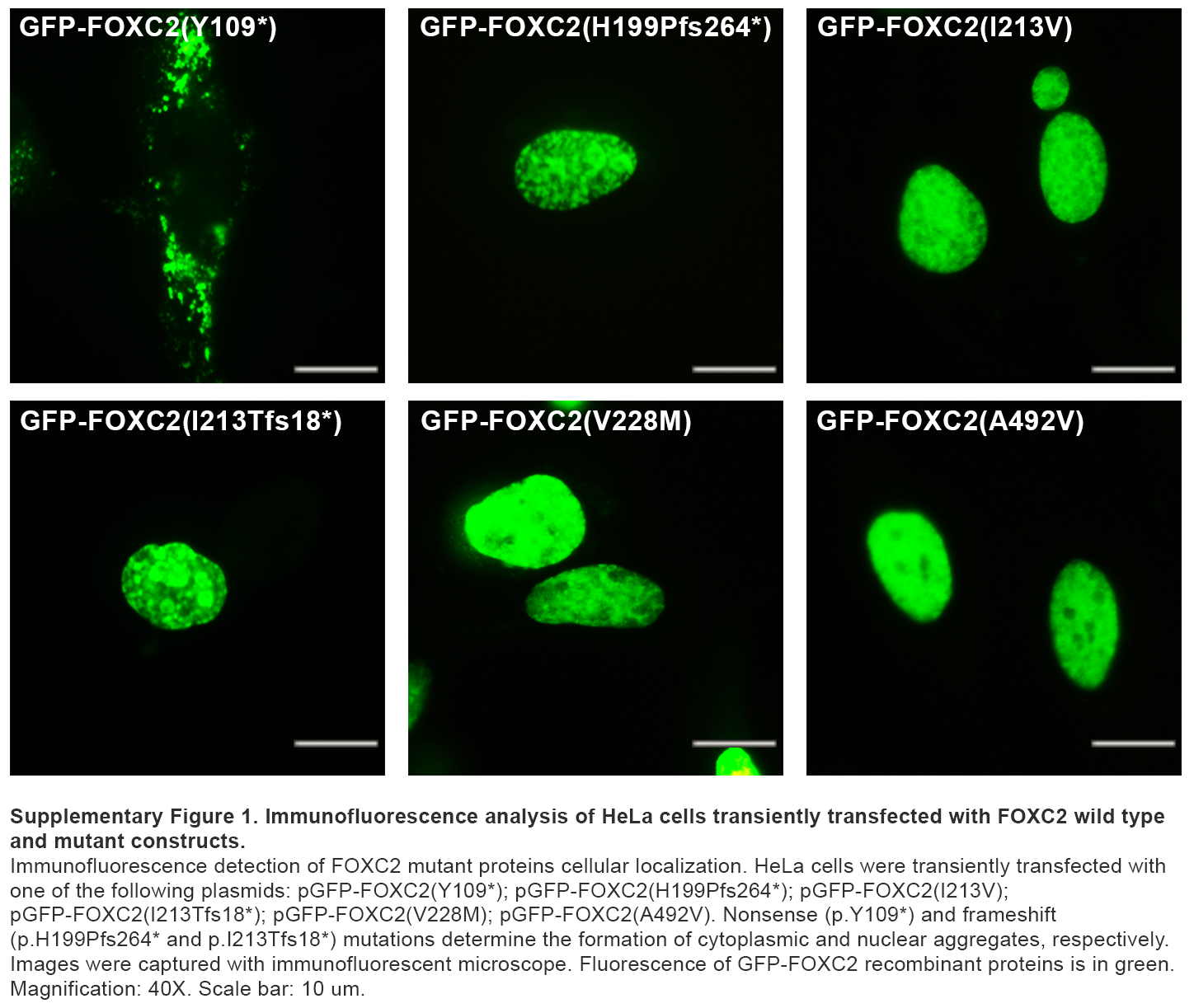

Supplement: Supplementary file 1 [file genes-12-00650-s001.zip › Supplementary Figure 1.tif]

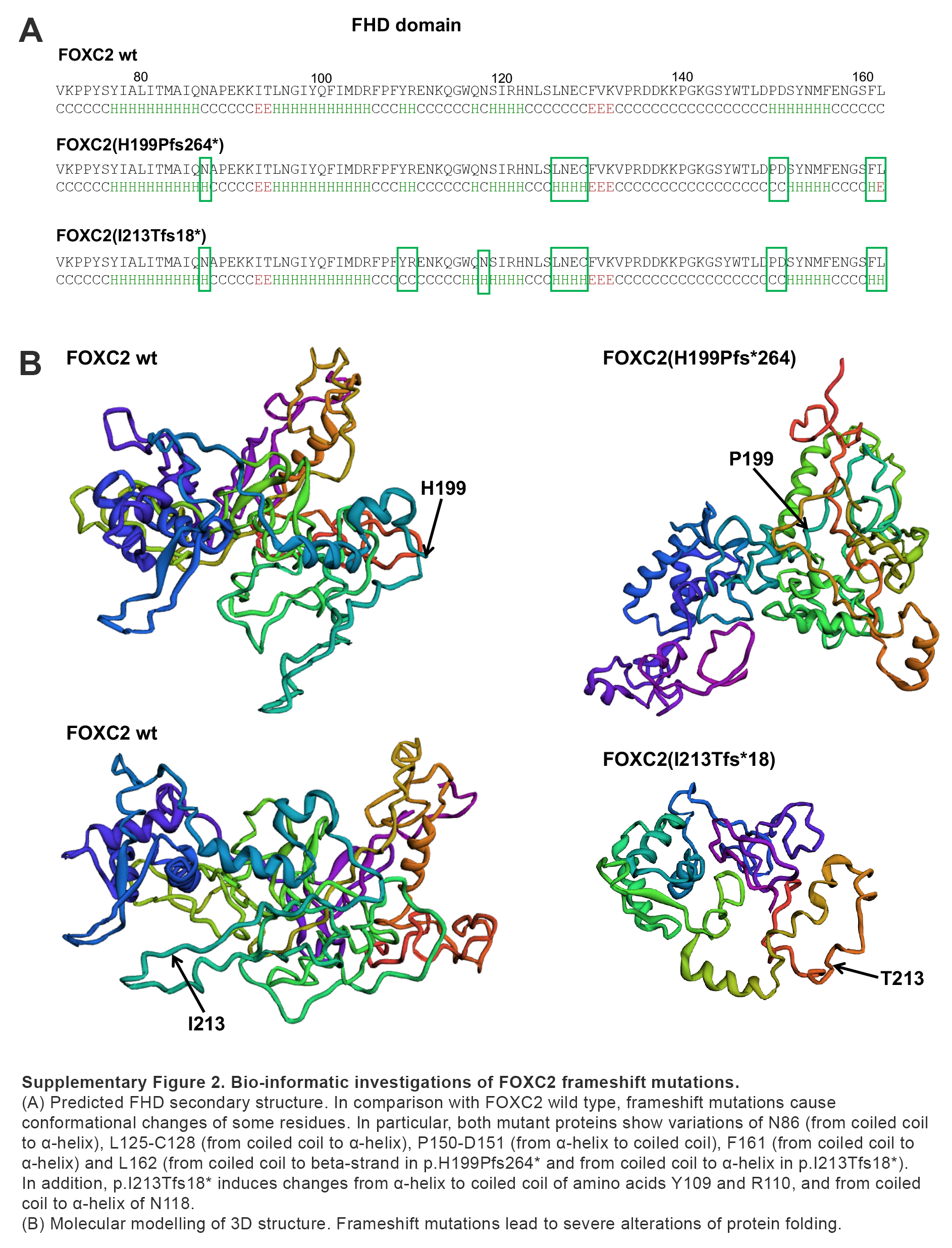

Supplement: Supplementary file 1 [file genes-12-00650-s001.zip › Supplementary Figure 2.tif]
